# The Biocompatibility Analysis of Artificial Mucin-Like Glycopolymers

**DOI:** 10.3390/ijms241814150

**Published:** 2023-09-15

**Authors:** P. Trosan, J. S. J. Tang, R. R. Rosencrantz, L. Daehne, A. Debrassi Smaczniak, S. Staehlke, S. Chea, T. A. Fuchsluger

**Affiliations:** 1 Department of Ophthalmology, University Medical Center Rostock, 18057 Rostock, Germany; 2 Biofunctionalized Materials and (Glyco) Biotechnology, Fraunhofer Institute for Applied Polymer Research IAP, 14476 Potsdam, Germany; 3 Institute of Materials Chemistry, Chair of Biofunctional Polymer Materials, Brandenburg University of Technology BTU, 01968 Senftenberg, Germany; 4 Surflay Nanotec GmbH, 12489 Berlin, Germany

**Keywords:** epithelial corneal cells, cornea, glycopolymers, mucin

## Abstract

The ocular surface is covered by a tear film consisting of an aqueous/mucin phase and a superficial lipid layer. Mucins, highly *O*-glycosylated proteins, are responsible for lubrication and ocular surface protection. Due to contact lens wear or eye disorders, lubrication of the ocular surface can be affected. Artificial glycopolymers which mimic natural mucins could be efficient in ophthalmic therapy. Various neutral, positively, and negatively charged mucin-mimicking glycopolymers were synthesized (n = 11), cultured in different concentrations (1%, 0.1%, and 0.01% *w*/*v*) with human corneal epithelial cells (HCE), and analyzed by various cytotoxicity/viability, morphology, and immunohistochemistry (IHC) assays. Six of the eleven glycopolymers were selected for further analysis after cytotoxicity/viability assays. We showed that the six selected glycopolymers had no cytotoxic effect on HCE cells in the 0.01% *w*/*v* concentration. They did not negatively affect cell viability and displayed both morphology and characteristic markers as untreated control cells. These polymers could be used in the future as mucin-mimicking semi-synthetic materials for lubrication and protection of the ocular surface.

## 1. Introduction

Contact lenses (CL), besides correcting refractive errors, help people live a more active life with fewer restrictions. They are also prescribed for aphakia, keratoconus, irregular cornea, Stevens–Johnson syndrome, Sjogren syndrome, or persistent epithelial effect [1,2,3,4,5]. Unfortunately, CL wear is associated with many complications like trauma, decreased corneal oxygenation, reduced corneal and conjunctival lubrication, stimulation of allergic and inflammatory responses, and infection. According to the investigation of Dumbleton, primary reasons for discontinuation of wearing the CL were discomfort (24%), dryness (20%), red eyes (7%), and expense (7%) [6]. CL overwear is also one of the risk factors for dry eye syndrome [7].

CL wear divides the tear film into two parts, pre- and post-lens layers [8]. It can affect biochemical and biophysical properties of the ocular surface microenvironment and lead to reduced tear stability [8]. The tear film is responsible for the lubrication and protection of the ocular surface; it is approximately 3 to 10 μL in volume, 3 μm thick, and secreted at a rate of 1 to 2 μL/min [9]. The pre-lens layer divided by a CL is 2 µm thick, 1µm thinner than the regular thickness [10].

The tear film is a heterogeneous complex of proteins, lipids, metabolites, and electrolytes. It consists of three layers; the inner mucin, middle aqueous, and outer lipid layer. Mucins can be membrane-associated and involved in signal transduction (mucins MUC 1, 4, and 16) or secreted, and are responsible for the protection, lubrication, and hydration of the ocular surface (MUC5AC, MUC7) [11,12,13]. Their structure consists of a polypeptide backbone predominantly made of serine, proline, glycine, and threonine, on which oligosaccharide side chains covalently bind via *O*-glycosidic bonds [14]. These chains are mostly built from compounds which determine their hydrophilic properties, like monosaccharide subunits, galactose, or sialic acids [15]. Understanding of mucins’ function and structure is reflected in their use as part of innovative functional biomaterials or as bio-inspiration in synthetic chemistry. The three main general methods used for the synthesis of the glycopolymers are: solid-phase synthesis of glycosylated amino acids, solution polymerization from glycomonomers, or post-polymerization glycosylation [16]. Glycopolymers have recently been used for drug, gene, and antigen delivery [17,18,19,20].

Mucin-mimicking glycopolymers exhibiting high hydrophilicity like natural mucins and higher stability due to their semi-synthetic nature have been synthetized lately [21]. Synthesis was performed via aqueous-based routes from *N*-acetylgalactosamine (GalNAc), lactose (Lac), or maltose (Mal). GalNAc belongs to O-linked glycans, the vast majority of oligosaccharides on natural mucins [22]. The disaccharides lactose and maltose consist of galactose or glucose which are also components found in natural mucins [23]. Mucins are naturally negatively charged by sulfate and, mainly, sialic acid groups. Therefore, our anionic glycopolymers were prepared by a free radical polymerization of glycomonomers and gained negative charges by one-step chemical sulfation. Cationic glycopolymers were prepared by an aqueous-based one-step synthesis of glycosylated branched polyethyleneimine (PEI). Branched PEI was used as a polymer backbone and its amines were employed for functionalization with saccharide’s and glycopolymer’s final positive charge [21].

The complementary charge of the mucin-mimicking glycopolymers allows their use in the Layer-by-Layer (LbL) self-assembly system. This system was invented 30 years ago and offers a simple adsorption of oppositely charged polyelectrolytes on surfaces [24]. This technique is efficient for fabricating coating systems like drug delivery systems with great versatility and control [25]. Recently, thin films of mucin-mimicking glycopolymers were fabricated by an LbL self-assembly system and tested as a scaffold for liposomes [21]. Active substances (e.g., drugs) can potentially be encapsulated in the liposomes or, in cases of charged drugs, directly immobilized in the LbL film [26]. Glycopolymer films, with and without liposomes, showed their stability in physiological pH and lipid vesicles integrity.

In this study, the effects of various types of mucin-mimicking glycopolymers on the viability, proliferation, morphology, and protein expression of the human corneal epithelial cells were studied, as well as their cytotoxic and cytostatic effects.

## 2. Results

### 2.1. Synthesis and Characterization of Glycopolymers

**Figure 1:**
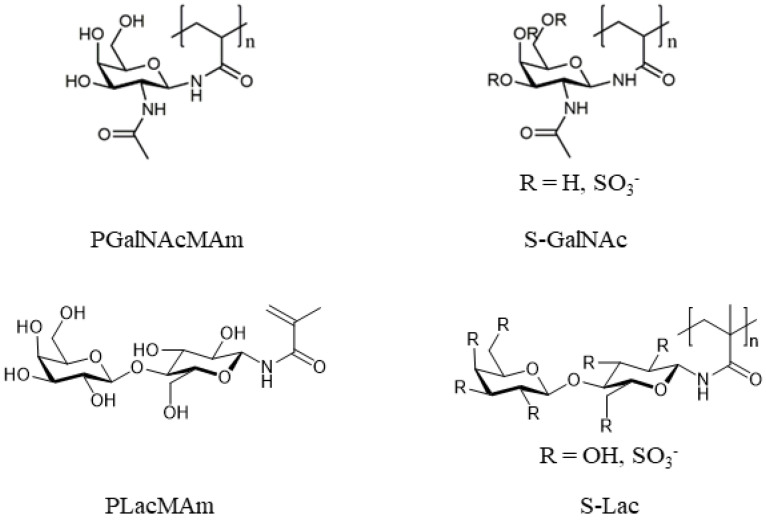
Chemical structures of neutrally and negatively charged glycopolymers.

We prepared glycopolymers of different charges as described in a previous work [21]. First, glycomonomers were synthesized from GalNAc or Lac by amination and consecutive methacrylation. The resulting glycomonomers, GalNAcMAm and LacMAm, were then polymerized separately to yield linear glycopolymers, named PGalNacMAm or PLacMAm (Figure 1).

To gain negative charges, the pending saccharides were sulfated (Figure 1). The sulfation method is a direct, one-step functionalization which ensures the homogeneous distribution of the charges on the polymer since the hydroxyl groups of the C-2 and C-6 positions are favored by the sulfation reaction. As the disacharide Lac offers more hydroxyl groups than GalNac, the sulfated Lac-based polymer (S-Lac) offers a higher functionalization degree and therefore a higher charge density than the GalNAc-based polymer (S-GalNAc). ^1^H NMR spectra of these glycopolymers confirmed their successful syntheses and are depicted in the Supplementary Information. GPC measurements revealed high values for the dispersity Đ (Table 1). This indicates side reactions, such as branching, during synthesis which can occur in uncontrolled free-radical polymerizations.

**Figure 2:**
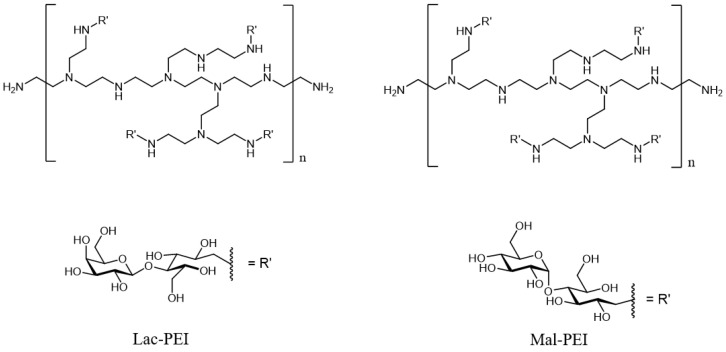
Chemical structures of cationic glycopolymers.

Cationic glycopolymers were prepared by the glycosylation of PEI via reductive amination. This method allows the one-step synthesis of positively charged glycopolymers as PEI consists of many amino groups which offer facile functionalization as well as positive charges (Figure 2).

A ^1^H NMR spectroscopy confirmed the successful glycosylation of PEI (Figures S3–S13). The GPC characterization showed a very high dispersity for Lac-PEI-750k, while the other glycosylated PEIs had a lower dispersity of 1.7–2.9. The high dispersity could be caused by possible boron residues from the synthesis as they could form boronate esters between the saccharides and, therefore, cause crosslinking of the polymers [21]. Generally, the measured molecular weight of the glycosylated PEIs is higher than the molecular weight of the commercial PEIs that were used for synthesis. This indicates the successful functionalization of PEI. One exception is Mal-PEI-750k as its determined molecular weight is significantly lower than 750 kDa. It has to be noted that the GPC calibration for the cationic polymers was performed with dextran standards with structures different from our glycopolymers. Hence, the GPC results can possibly differ from actual values. The functionalization degree of PEI was determined by its sugar content via phenol-sulfuric acid assay. All Lac-PEIs exhibit similar sugar contents, while Mal-PEI has a sugar content about twice as high.

### 2.2. Cytotoxicity/Viability Tests

Various mucin mimicking glycopolymers with different surface charges were synthe-tized (Table 2). All of them were tested with cytotoxicity and viability assays. Based on these outcomes, the two of each type (neutrally, positively and negatively charged) glyco-polymers were selected and tested with subsequent analyses. Results of unselected glyco-polymers (PLacMam-1, Lac-PEI-25k, Lac-PEI-750k, Mal-PEI-750k and S-Lac-3) were added to the Supplementary information (Figures S1 and S2).

**Figure 3:**
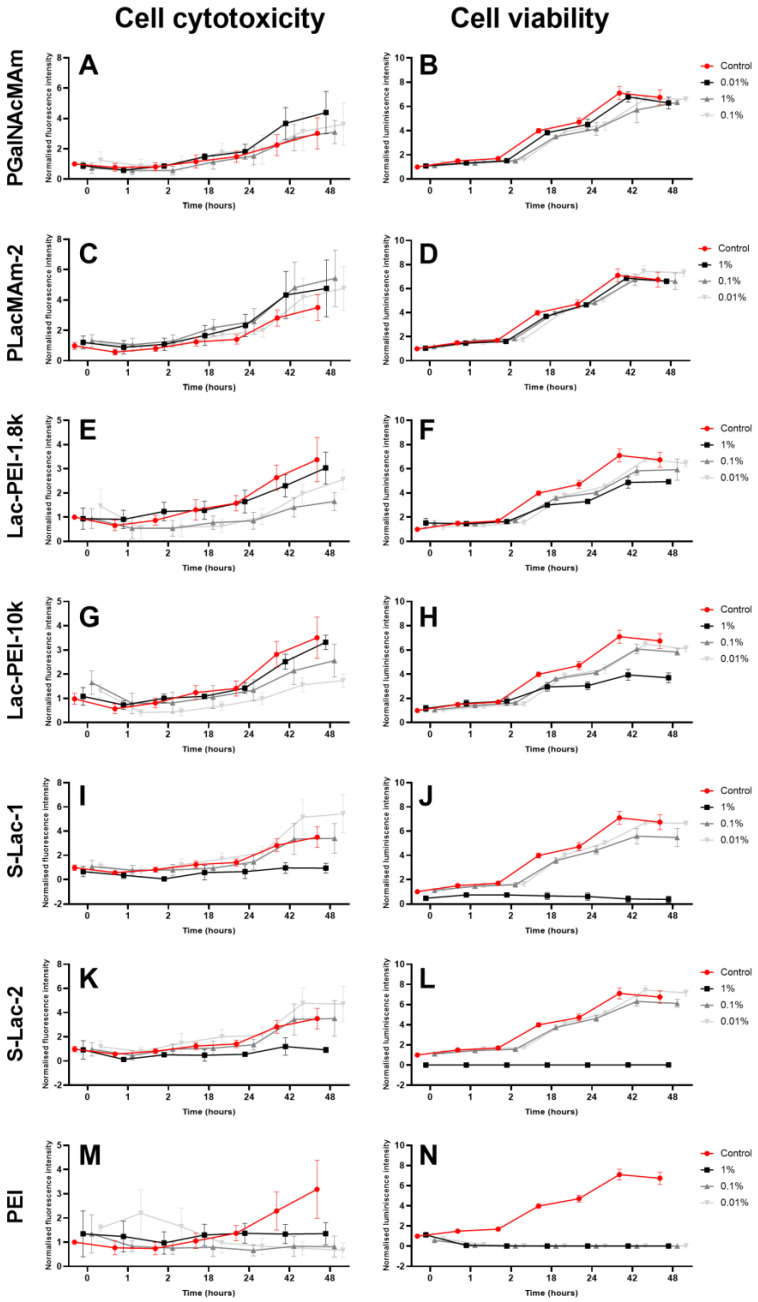
The cytotoxicity (**A**,**C**,**E**,**G**,**I**,**K**,**M**) and viability (**B**,**D**,**F**,**H**,**J**,**L**,**N**) assays of cultured HCE itself (control), with different glycopolymers (in 1, 0.1, and 0.01% *w*/*v* concentration) and PEI (MW 750k). The intensity of luminescence (viability; *y* axis) and fluorescence (cytotoxicity; *y*-axis) was measured at various time points (0–48 h; *x*-axis) by Microplate Reader. The data were normalized to control at time point 0. Interleaved symbols were used for every time point in the graphs.

**Figure 4:**
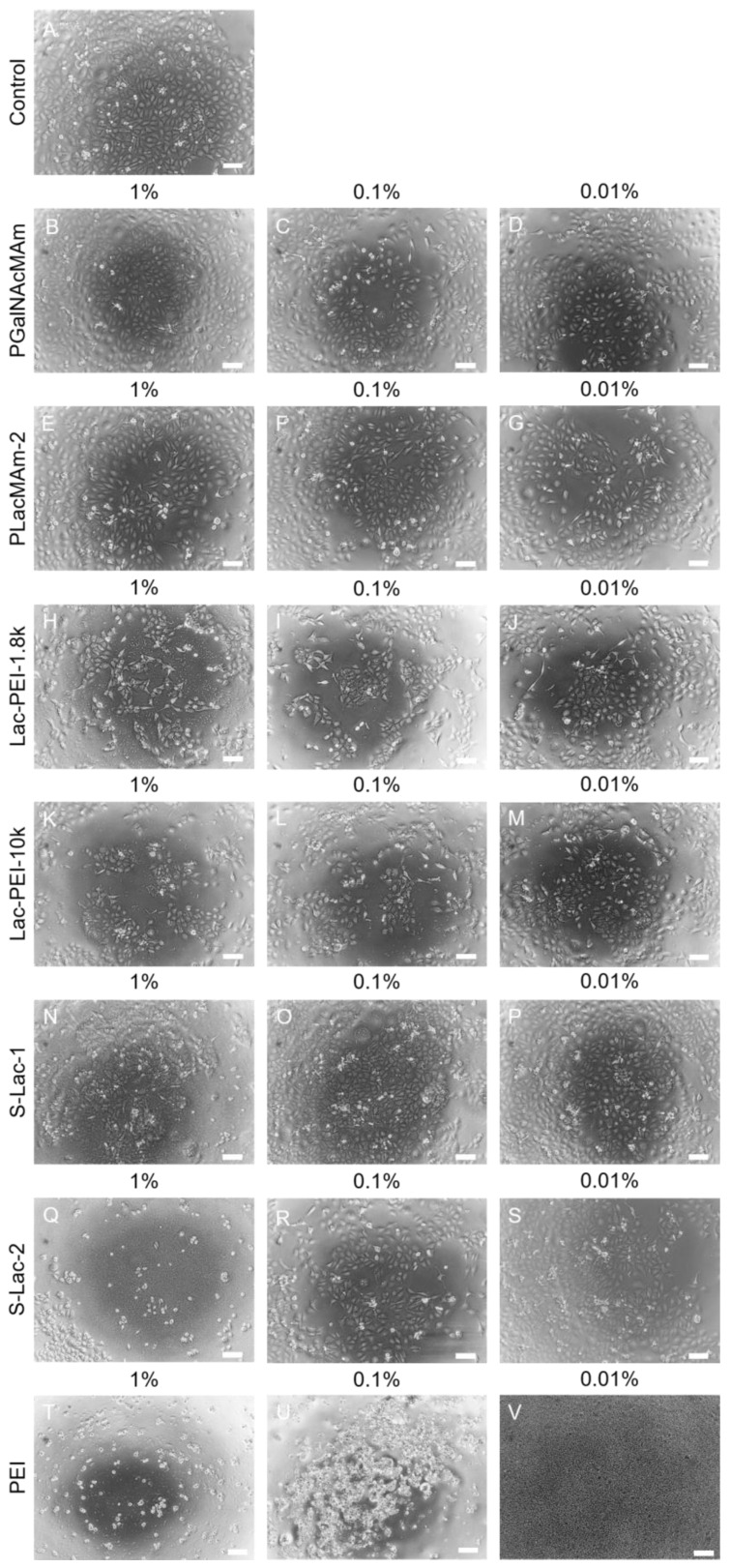
Morphology of cultured HCE itself (control; (**A**)), with PGalNAcMAm (**B**–**D**), PLacMAm-2 (**E**–**G**), Lac-PEI-1.8k (**H**–**J**), Lac-PEI-10k (**K**–**M**), S-Lac-1 (**N**–**P**), S-Lac-2 (**Q**–**S**) and PEI (MW 750k; (**T**–**V)**) in 1, 0.1 and 0.01% *w*/*v* concentration after 48 h. Light microscopy, scale bars represent 100 μm.

The culture of HCEs with all neutral glycopolymers (PGalNAcMAm, PLacMAm-1 and PLacMAm-2) showed a similar level of cytotoxicity as the control cells. Only a minor increase without statistical significance was measured after 42 and 48 h of culture, which could be caused by cell confluence (Figure 3A,C and Figure S1A). The results from the viability measurement showed no difference between cell cultures with both neutral glycopolymers in all concentrations and control cells (Figure 3B,D and Figure S1B). Moreover, the morphology of treated cells was about the same as in control cells (Figure 4A–G andFigure S2A–D). The two batches of PLacMam were synthetized for this project, and results with both of them (PLacMAm-1 and PLacMAm-2) were very similar. For subsequent experiments only PLacMAm-2 was used.

The five various positively charged glycopolymers were synthetized (Lac-PEI-1.8k, Lac-PEI-10k, Lac-PEI-25k, Lac-PEI-750k and Mal-PEI-750k). They were different in type of used sugars for glycosylation and in molecular weight of PEI, which was used as a polymer backbone. The co-culture of HCE with Lac-PEI-1.8k and Lac-PEI-10k showed the same level of cytotoxicity when 1% *w*/*v* of glycopolymers was used, compared to the control cells (Figure 3E,G). The culture with a lower w/v concentration of mucin-mimicking glycopolymers (0.1, 0.01%) showed an even lower cytotoxicity as the control. Cultures of HCE with Lac-PEI-25k, Lac-PEI-750k and Mal-PEI-750k showed no increase of cytotoxicity in all glycopolymers concentrations compared to control cells (Figure S1C,E,G). Cultures with all positively charged glycopolymers in 0.1 and 0.01% concentrations had similar viability as control cells. With a higher concentration of glycopolymers (1% *w*/*v*), lower values of cell viability were measured compared to the controls (Figure 3F,H and Figure S1D). The difference in viability of HCE between the used w/v concentrations of Lac-PEI-1.8k and Lac-PEI-10k was also visible in the number of cells via light microscopy; a lower concentration of glycopolymers and higher number of cells (Figure 4H–M). Measurement of cultures with Lac-PEI-750k and Mal-PEI-750k showed the lower viability of cells the higher concentrations of glycopolymer were used (Figure S1F,H). Together with no increase of cytotoxicity, the cytostatic effect was observed here. Lower viability, change of morphology and number of dead cells were also observed by light microscopy (Figure S2H–M). Molecular weight of PEI was decisive in selection of positively charged glycopolymers for subsequent tests.

The three types of negatively charged glycopolymers were synthetized (S-Lac-1, S-Lac-2 and S-Lac-3). The culture of HCE with 1% *w*/*v* of all negatively charged glycopolymers led to a completely decreased cell viability (Figure 3J,L and Figure S1J), also confirmed via light microscopy (Figure 4N,Q and Figure S2N–P). The cytotoxic effect was not detected (Figure 3I,K and Figure S1I). Similar cytotoxicity, viability, and cell morphology were monitored in the HCE cultures as in the control cells when lower concentrations of S-Lac-1 and S-Lac-2 were used (0.1, 0.01% *w*/*v*) (Figure 3I–L, Figure 4A,N–S, Figures S1I,J and S2A,N–P). Results of S-Lac-3 glycopolymer were likewise in other two negatively charged glycopolymers (S-Lac-1 and S-Lac-2). The difference between them is in sulfation degree. In contrast to other two, the S-Lac-3 glycopolymer was more difficult to dissolve and therefore was not used for subsequent testing.

Cell viability, cytotoxicity, and morphology were strongly affected by the co-culture of cells with all of the *w*/*v* concentrations of PEI (MW 750k) (Figure 3M,N and Figure 4T–V).

### 2.3. Determination of the Metabolic Activity

**Figure 5:**
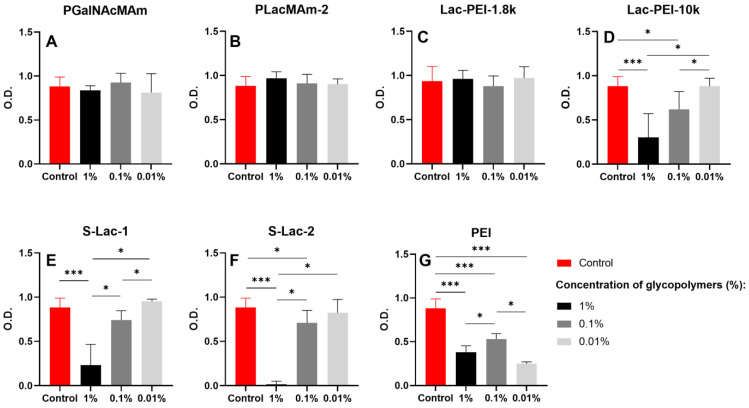
Comparison of the metabolic activity of the cultured HCE itself (control), with PGalNAcMAm (**A**), PLacMAm-2 (**B**), Lac-PEI-1.8k (**C**), Lac-PEI-10k (**D**), S-Lac-1 (**E**), S-Lac-2 (**F**) and PEI (MW 750k; (**G**)) in 1, 0.1 and 0.01% *w*/*v* concentration after 48 h. WST-8 reagent was added to the cell cultures for 4 h to form formazan. The absorbance was measured at a wave-length of 450 nm. * *p* < 0.05, *** *p* ≤ 0.001.

Similar metabolic activity was measured in cultures of HCE with all w/v concentrations of neutral mucin-mimicking glycopolymers compared to the controls (Figure 5A,B). No difference was measured between the values of the metabolic activity of control cells and cells cultured with various *w*/*v* concentrations of Lac-PEI-1.8k (Figure 5C). However, the metabolic activity was significantly decreased in cell cultures with 1% of Lac-PEI-10k compared to the control and to cultures with 0.01% of Lac-PEI-10k (*p* ≤ 0.001, *p* < 0.05, respectively) (Figure 5D). The HCE cultures with 0.1% of Lac-PEI-10k had significantly less metabolically active cells as the control and cultures with 0.01% of the same glycopolymer (both *p* < 0.05). Cells cultured with 0.01% of Lac-PEI-10k had the same metabolic activity as the control cells.

The measurement of the metabolic activity of cells cultured with 0.01% of both negatively charged mucin-mimicking glycopolymers showed no difference compared to the controls. On the contrary, cultures with 1% of S-Lac-1 and S-Lac-2 had a decreased activity to the control, 0.1 and 0.01% of glycopolymers cultures (*p* ≤ 0.001, *p* < 0.05, *p* < 0.05, respectively) (Figure 5E,F). All the experiments of HCEs with various concentrations of PEI (MW 750k) led to decreased metabolic activity, compared to the controls (Figure 5G).

### 2.4. Live/Dead Assay

**Figure 6:**
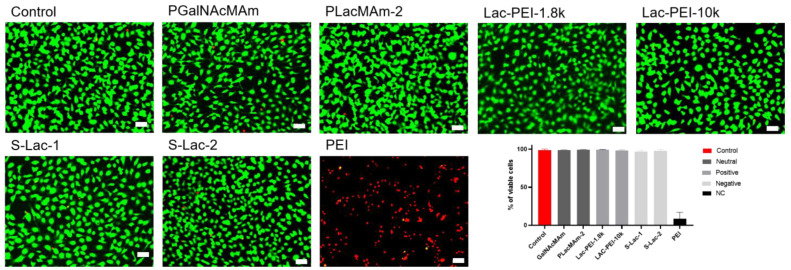
Comparison of the percentage of viable cultured HCE itself (control) with different glycopolymers (0.01% *w*/*v* concentration) and PEI (MW 750k). A Live-Dead assay was applied after 48 h. Live cells were stained with Calcein (green) and dead cells with Ethidium homodimer-1 (red). The scale bar presented in each image is 50 µm.

Cultures of HCE with 0.01% of neutral, positively, and negatively charged glycopolymers had the same percentage of viable cells after 48 h compared to the controls. Using PEI (MW 750k) with the cells led to a significant decrease in viability (Figure 6).

### 2.5. Proliferation Analysis

**Figure 7:**
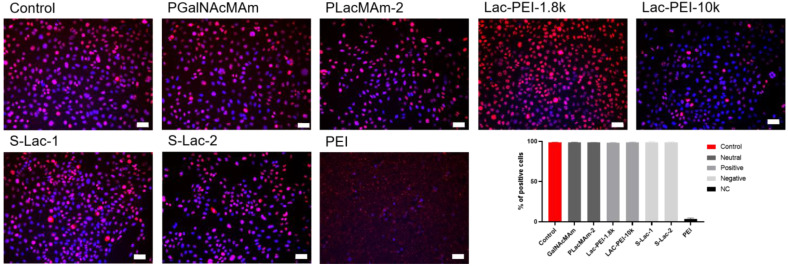
Immunostaining of proliferation marker Ki-67 (red) in cultured HCE itself (Control) with different glycopolymers (in 1, 0.1, and 0.01% *w*/*v* concentration) and PEI (MW 750k). Cell nuclei were counterstained with DAPI (blue). The scale bar represents 50 μm.

Immunocytochemistry of the samples for the proliferation marker Ki-67 showed no difference between the cultures with 0.01% of select mucin-mimicking glycopolymers and the controls. The samples had a similar percentage of Ki-67 positive cells with no significant difference. The decrease in the percentage of positive cells was observed in cells cultured with 0.01% of PEI (MW 750k) for 48 h, compared to all other samples (*p* ≤ 0.001) (Figure 7).

### 2.6. Analysis of Epithelial Markers

**Figure 8:**
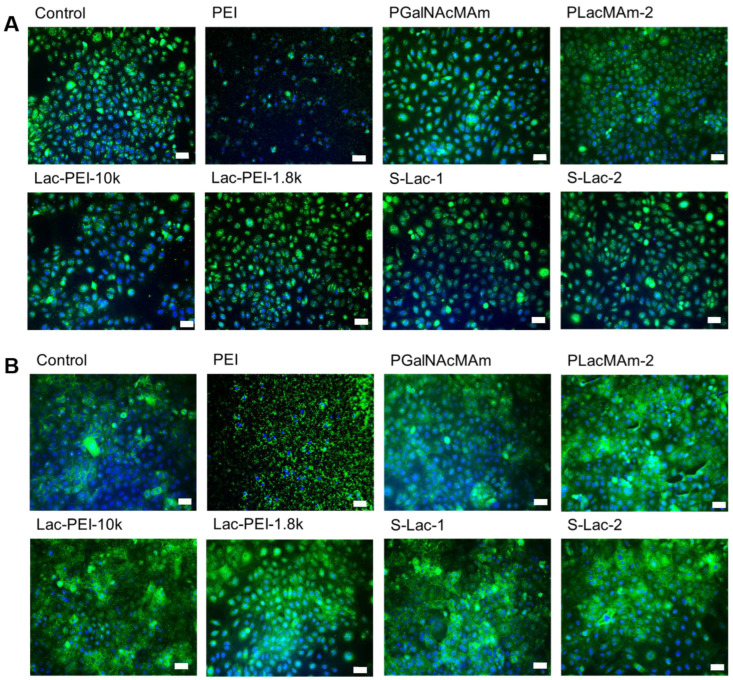
Immunostaining of markers of HCE (Pax6 (**A**); ABCG2 (**B**)) in cultured HCE itself (control) with different glycopolymers (in 1, 0.1, and 0.01% *w*/*v* concentration) and PEI (MW 750k). Cell nuclei were counterstained with DAPI (blue). The scale bar represents 50 μm.

The cultures with 0.01% neutral, positively, and negatively charged glycopolymers showed the same intensity and positivity of the staining for protein Pax6 as control cells (Figure 8A). Similarly, the use of glycopolymers did not lead to a change in expression of ABCG2, compared to the control (Figure 8B). Cultures with 0.01% of PEI (MW 750k) led both ICC to negative and unspecific staining (Figure 8A,B).

## 3. Discussion

Glycopolymers display interesting characteristics in cell–cell adhesion, protein interaction, targeted drug delivery, tissue engineering, and the synthesis of biocompatible materials [27,28,29]. We synthetized several mucin-mimicking glycopolymers with various charges. The use of these glycopolymers in an LbL self-assembly system and their subsequent preclinical study has to forego biocompatibility testing as biocompatibility is one of the main prerequisites for the clinical use of biomaterials. The influence of toxic agents derived from biomaterials can lead to a loss of cell adhesion, changes in cellular morphology, reduced cellular proliferation or metabolic activity, and cell death. A corneal epithelial cell line was used to test the biocompatibility of the glycopolymers and various methods for estimating cytotoxicity, viability, metabolic activity, proliferation, or cell phenotype.

By the first cytotoxicity/viability measurements, we were able to select the six potential biocompatible mucin-mimicking glycopolymers. The test of neutral glycopolymers showed the same biocompatibility as control cells. Unfortunately, biomaterials without a surface charge could not be used for the LbL self-assembly system. Therefore, the glycopolymers with positive or negative charges distributed all over the glycopolymer were synthesized, in contrast to glycopolymer coating which stabilized superparamagnetic nanoparticles and can attach the surface only by electrostatic interaction between the carboxylic acid terminal of the glycopolymer and the iron oxide surface [30]. Glycopolymers with negative charges showed the cytostatic but not the cytotoxic effect when 1% *w*/*v* concentration was used. The cytostatic effect was determined due to a lower viability but no change in signal for cytotoxicity as a result of compromised membrane integrity, indicating growth inhibition and not cytotoxicity. The biocompatibility of glycopolymers depends on various factors, like polymer morphology, its concentration, or length of incubation [31]. The use of lower concentrations of negatively charged glycopolymers in the cell cultures led to increased viability and showed their biocompatibility. We hypothesized, that decrease in viability with higher glycopolymer concentration could be associated with sulfate groups. It was observed that keratinocytes had a dose-dependent decrease in viability under the effect of sodium dodecyl sulfate [32].

On the contrary, cationic materials should be able to better adhere to negatively charged ocular surfaces. A positively charged poly(l-histidine) coating of the nanoparticles can bind to the cellular membrane surface and enables cell/tissue permeability [33,34]. Moreover, the poly(l-histidine) surface coating of the nanocages led to high corneal penetration capability and the effective release of the drugs in the chemical eye injury [35]. We used PEI as a backbone in the synthesis of positively-charged mucin-mimicking glycopolymers. PEI is one of the most efficient non-viral gene delivery vectors for the transfection of cells [36]. However, it also has a cytotoxic impact on target cells through the induction of apoptosis/necrosis [37]. Factors like molecular weight or composition can affect the transfection ability and cytotoxicity. It was shown that branched PEI had a higher transfection rate than linear PEI, but could also initiate apoptosis [38]. PEI with lower molecular weight, such as 800 Da or 2000 Da, had a lower cytotoxicity than 25 kD [31,39]. We used PEI with various molecular weights (1.8 kD–750 kD) for the synthesis of cationic glycopolymers, and our results also confirmed a lower cytotoxic effect of PEI when a lower molecular weight was used. To improve PEI biocompatibility, modifications, including the physical or chemical addition of poly (ethylene glycol), folic acid, or heparin, can be used [40,41]. It was also shown that the biocompatibility of the star-shaped glucosamine-functionalized polymers can be optimized by modifying the molar ratio of polylysine to the glycopolymer’s arms [42]. We established the functionalization of PEI with lactose, maltose, or glucuronic acid for the positively charged mucin-mimicking glycopolymers to increase their biocompatibility. Besides molecular weight, the cationic charge density, concentration, and incubation time of the polymer have an effect on the cell viability. Similarly to our experiments, the cytotoxicity of the cationic polymers, including PEI, decreased in relation to decreasing concentrations and incubation times [43].

The measurement of metabolic activity via CCK-8 assay was used to confirm a previous cytotoxicity/viability evaluation. The 0.01% *w*/*v* concentration of selected glycopolymers was specified as biocompatible according to HCE culture experiments. As shown by others, the lower the polymer concentration in cell cultures, the higher the viability of 293FT and HeLa cells [44]. Similarly, we found no decrease in the percentage of viable cells after culture with the lowest concentration of glycopolymers. Moreover, the ability to proliferate was also not affected by the mucin-mimicking glycopolymers, regardless of the surface charge. Notably, the cytotoxic effect of PEI can be at least partially diminished by glycosylation without impeding the overall charged nature of the polymer.

Beside cell cytotoxicity, viability, or proliferation, it is important that cells do not change morphology or the expression of their proteins as these are typical markers for the specific cell type. Immortalized HCEs have the same morphology as corneal epithelial cells and express the same typical markers as PAX-6 and ABCG2 [45,46]. Our HCEs cultured with all of the select glycopolymers exhibit the same morphology and protein expression as control cells. The co-culture of cells with PEI led to changes in morphology, cell death, and indemonstrable and nonspecific PAX-6 and ABCG2 protein expression. A similar observation was made in cells stained with vimentin after culture with N-acetylglucosamine-PEI complexes [44].

In conclusion, it could be shown that select glycopolymers (PGalNAcMAm, PLacMAm-2, Lac-PEI-1.8k, Lac-PEI-10k, S-Lac-1, and S-Lac-2) had no cytotoxic effect on HCE cells in the 0,01% *w*/*v* concentration. The glycopolymers had no negative impact on cell viability and displayed morphology and characteristic markers as untreated control cells.

## 4. Materials and Methods

### 4.1. Materials and Methods for Artificial Glycopolymer Synthesis

All chemicals listed in Table 3 were purchased from commercial sources and used as received if not stated otherwise.

Nuclear magnetic resonance (NMR) spectra were recorded on a Bruker Avance 300 MHz Spectrometer (Billerica, MA, USA). ^1^H NMR samples were dissolved in D_2_O. Elemental analysis was carried out using FlashEA 1112 CHNS/O Automatic Elemental Analyzer (Thermo Fisher Scientific; Waltham, MA, USA) to determine the sulfation degree of sulfated Glycopolymers. Gel permeation chromatography (GPC) was performed using a WGE SEC-3010 system with an RI detector and chromatography columns TSK Gel and PSS Suprema, respectively. Flow rate was 1 mL min^−1^. For neutral and anionic polymers, the eluent was 0.07 m disodium phosphate buffer and calibration was performed with polyacrylic acid standards. For cationic polymers, the eluent was aqueous potassium chloride (0.2 mol·L^−1^) with formic acid (0.2 mol·L^−1^) and calibration was conducted with dextran standards.

### 4.2. Synthesis of Glycopolymers

Synthesis of neutrally charged glycopolymers PGalNAcMAm and PLacMAm: Glycopolymers was prepared by free radical polymerization of glycomonomers, namely GalNAcMAm and LacMAm, which are based on a methacrylamide (MAm) functionalized with GalNAc or Lac, to yield neutrally charged glycopolymers carrying GalNAc or Lac moieties (Table 2). Their syntheses and characterization are described in previous publications [21,25]. Briefly, GalNAcMAm and ABCVA (2 mol%) were dissolved in Milli-Q water (0.4 mmol·mL^−1^) and purged with nitrogen for at least 30 min. Afterward, the reaction vessel was submerged in an 80 °C oil bath to start polymerization. The reaction was allowed to proceed for about 2 d. The polymer was purified by dialysis against deionized water before freeze drying. A white fluffy solid (PGalNAcMAm) (76%) was obtained. For the preparation of PLacMAm, LacMAm and ABCVA (2 mol%) were dissolved in Milli-Q water (0.3 mmol·mL^−1^) and followed the same procedure as above for the synthesis of PGalNAcMAm. Two different batches were prepared, PLacMAm-1 and PLacMAm-2, and a white fluffy solid (~82%) was yielded.

Synthesis of the anionic sulfated glycopolymer S-Lac: To gain negative charges, neutrally charged glycopolymer PLacMAm was chemically sulfated similar to the procedure of previous publications [21,26]. Three different batches were prepared to yield S-Lac-1, S-Lac-2, and S-Lac-3 (Table 2). Briefly, PLacMAm was suspended in dry DMF (10 mg·mL^−1^ for S-Lac-1 and S-Lac 2; 20 mg·mL^−1^ for S-Lac-3) in a dried Schlenk flask under nitrogen atmosphere. The suspension was stirred for 18 h before chlorosulfonic acid (3.3 equiv. per OH for S-Lac-1 and S-Lac 2; 4 equiv. per OH for S-Lac-3) was added. The reaction mixture was heated to 50 °C and stirred for further 5 h. Afterwards, saturated NaOAc-EtOH solution (15 mL) was added and the precipitate was centrifuged (14,000 rpm, 10 min). Sediment was washed and centrifuged with EtOH again. Then, it was dissolved in little deionized water and dialyzed against deionized water. After freeze drying, a white fluffy solid was obtained. A sulfation degree of 0.75 sulfate groups per monomer unit was achieved for S-Lac-1, 1.16 for S-Lac-2 and 1.68 for S Lac 3. A sulfation degree of 0.75 sulfate groups per monomer unit was achieved for S-Lac-1, 1.16 for S-Lac-2 and 1.68 for S-Lac-3. Anal. Found (S-Lac-1): C 33.31, H 5.71, N 2.55, S 4.38. Anal. Found (S-Lac-2):C 30.31, H 5.14, N 2.41, S 6.42. Anal. Found (S-Lac-3):C 28.18, H 4.63, N 2.43, O 43.74, S 9.34.

Synthesis of cationic glycopolymers Lac-PEI and Mal-PEI: The synthesis and characterization of Lac and Mal functionalized PEI is described in previous publication [21]. Here, PEI of different molecular weights was employed. The respective molecular weight of the polymer backbone is annotated in Da behind each sample name (e.g., “Lac-PEI-10k” describes PEI of 10 kDa that was glycosylated with Lac). For the synthesis of Lac-PEI-750k, 10 g of PEI solution (50 wt%), lactose monohydrate (27 g, 75 mmol, 7 equiv. per monomer unit), MeOH (35 mL), and Na2B4O7 solution (50 mL, 50 mM) were stirred at 60 °C until completely dissolved. Subsequently, the reaction mixture was allowed to cool and the pH was adjusted to pH 3 with formic acid. NaCNBH3 (16.6 g, 264 mmol, 3.5 eq per lactose) was suspended in MeOH (15 mL, 1.1 g·mL^−1^) before adding it to the reaction mixture in portions. The reaction was allowed to proceed at 60 °C overnight. After reaction, MeOH was removed by rotary evaporation. Any precipitate was filtered with Kimtech Science^®^ precision wipes (Roswell, GA, USA). The yellow solution was dialyzed against deionized water and freeze dried to obtain a white solid. The preparation of the other glycosylated PEIs was conducted according to the procedure above with variation in the used amounts of lactose and NaCNBH3 (Table 2 and Table 4).

### 4.3. Phenol-Sulfuric Acid Assay for Determination of the Sugar Content of Glycosylated PEI

The phenol-sulfuric acid assay was performed following previous publication [47]. Glycopolymers were dissolved in water in three different concentrations (0.3, 0.6, and 1.0 mg·mL^−1^ for Lac-PEI; 0.15, 0.3, and 0.6 mg mL^−1^ for Mal-PEI). Then, 50 μL of sample was thoroughly mixed with sulfuric acid (150 μL). Afterwards, phenol (30 μL, 5%) was added and mixed. The solution was incubated at 90 °C for 5 min, cooled in a water bath for further 5 min, then was transferred into a 96-well plate (Carl Roth, Karlsruhe, Germany) and the absorption at 490 nm was measured. Calibration with lactose and maltose were used for calculating the total sugar amount.

### 4.4. Cell Culture

SV40-transformed human corneal epithelial cells (HCE) [45] were cultured in Dulbecco’s modified Eagle’s medium (DMEM)/Nutrient mixture F-12 Ham (Sigma-Aldrich) containing 10% fetal bovine serum (PAN-Biotech, Aidenbach, Germany) and 1% of 100× Antibiotic-Antimycotic (Thermo Fisher Scientific) in 75 cm^2^ tissue culture flasks (TPP, Trasadingen, Switzerland) at 37 °C in an atmosphere of 5% CO_2_; after this, referred to as medium. The medium was changed every 2 days. After reaching at least 90% confluence, adherent cells were harvested by 7 min incubation with 1 mL of TrypLE Express (Gibco, Grand Island, NY, USA) and, after centrifugation, used in specific concentrations (cell number) in the following experiments.

### 4.5. Cytotoxicity/Viability Tests

HCEs were seeded at a concentration of 5 × 10^3^ cells/well in 96-well plates and allowed 4 h for surface attachment. Afterwards, a medium with different artificial mucin-mimicking glycopolymers (in the 1%, 0.1%, and 0.01% final *w*/*v* concentration) was added together with the reagents for cytotoxicity/viability assays according to the manufacturer’s instructions. Measurement of changes in membrane integrity as a result of cell death was performed by CellTox™ Green Cytotoxicity Assay (Promega, Madison, WI, USA) and the determination of the number of viable cells was measured via RealTime-Glo™ MT Cell Viability Assay (Promega). Both assays were used together in each well and real-time repeatedly measured at certain time points (0, 1, 2, 18, 24, 42, and 48 h). Cytotoxicity was determined via increased fluorescence and viability according to measurements of luminescence using a GloMax^®^ Discover Microplate Reader (Promega). Cells cultured without glycopolymers were used as control and the HCEs co-cultured with PEI (MW 750k) as negative control (NC). The blank was subtracted for each concentration and type of glycopolymers individually.

Cell growth, confluence, and cell morphology were evaluated during the culture under an inverted microscope (VWR Visiscope IT405H; VWR, Radnor, PA, USA) and images were taken after 1, 24, and 48 h (images after 1 and 24 h are not shown).

### 4.6. Determination of Metabolic Activity

Metabolic activity of living cells was determined by Cell Counting Kit-8 (CCK-8) (Sigma-Aldrich). HCEs were seeded at a concentration of 5 × 10^3^ cells/well in a 96-well plate, 4 h were allowed for surface attachment. Afterwards, a medium with different artificial mucin-mimicking glycopolymers (in the 1%, 0.1%, and 0.01% final *w*/*v* concentration) was added and cultured at 37 °C in an atmosphere of 5% CO_2_. After 48 h, the medium was changed to the colorless DMEM medium (Gibco). CCK-8 reagent was added to each well (10 μL/100 μL of medium) and plates were incubated for another 4 h to form formazan. Absorbance was measured using a GloMax^®^ Discover Microplate Reader (Promega) at a wavelength of 450 nm.

### 4.7. Live/Dead Assay

HCEs were seeded at a concentration of 20 × 10^3^ cells/150 µL on Thermanox coverslips (Thermo Fisher Scientific) placed in 24-well plate. Cells were seeded in the middle of the coverslip and allowed to attach to the surface for 4 h. Afterwards, a medium with different artificial mucin-mimicking glycopolymers (0.01% final *w*/*v* concentration) was added and cultured at 37 °C in an atmosphere of 5% CO_2_ for 48 h. After that, the medium was removed and HCEs were washed with PBS. Coverslips were placed on the drop of PBS on parafilm with the cells down. The Live/Dead mixture of Ethidium Homodimer-1 and calcein were prepared according to the manufacturer’s instruction (Invitrogen, Carlsbad, CA, USA). Cells were stained with the mixture for 40 min and covered against the light. Subsequently, coverslips were washed 3 times with PBS, mounted with the aquatex mounting media (Merck, Rahway, NJ, USA), and immediately examined by fluorescence microscope (upgraded Zeiss Axioskop 40, Oberkochen, Germany). Pictures from various parts of coverslips were taken and evaluated.

### 4.8. Immunocytochemistry

Cells for immunocytochemistry (ICC) were cultured on Thermanox coverslips with the same procedure as for the Live/Dead assay described above. After 48 h culture, HCEs were fixed in 4% paraformaldehyde for 20 min at room temperature. Afterwards, cell membranes were permeabilized for 10 min with 0.3% Triton X-100 (Sigma Aldrich), followed by a 1 h blocking by 2.5% BSA/0.3% Triton X-100 diluted in PBS. Samples were incubated at room temperature with primary antibodies (according to Table 5) diluted in 0.1% BSA/0.3% Triton X-100 in PBS. Cells were then rinsed three times in 0.5% Tween 20 and incubated for 1 h at room temperature with the secondary antibodies (according to Table 5) diluted in 0.1% BSA/0.3% Triton X-100 in PBS. After rinsing three times in 0.5% Tween 20, followed by rinsing in PBS, cells were mounted with VectaShield-DAPI (4′6-diamidino-2-phenylindole) (Vector Laboratories, Burlingame, CA, USA) to counterstain nuclear DNA. Cell samples were examined by fluorescence microscopy at ×200 magnifications. The percentage of positive cells was calculated from pictures of different areas of each sample.

### 4.9. Statistical Analysis

Statistical analysis was performed with GraphPad Prism (GraphPad 9 Software, La Jolla, CA, USA). Descriptive statistics are reported as N (number of values), mean ± SD, or the median with quartile range. Data sets were analyzed by Mann–Whitney U nonparametric test. *p*-values < 0.05 were considered statistically significant.

## Figures and Tables

**Table 1:** Characterization of glycopolymers.

Glycopolymer	M_w_ [g/mol]	M_n_ [g/mol]	Đ	Sulfation Degree [Sulfate/Monomer Unit]	Sugar Content [wt%]
PGalNAcMAm	89,000	4600	19	—	—
PLacMAm-1	136,000	1900	72	—	—
PLacMAm-2	43,000	1800	25	—	—
S-Lac-1	199,000	9500	21	0.75	—
S-Lac-2	116,000	3100	37	1.16	—
S-Lac-3	90,000	4800	19	1.68	—
Lac-PEI-1.8k	7500	4500	1.7	—	12.3
Lac-PEI-10k	16,000	9000	1.7	—	14.6
Lac-PEI-25k	42,000	14,000	2.9	—	13.6
Lac-PEI-750k	4,830,000	20,000	242	—	16.2
Mal-PEI-750k	2400	1100	2.2	—	32.4

**Table 2:** The list of artificial glycopolymers. Glycopolymers in bold were selected after cytotoxicity/viability tests for subsequent analysis in this study. The cytotoxicity/viability results of the unselected glycopolymers were added in the Supplementary Information.

Neutrally Charged Glycopolymers	
**Poly(N-acetylgalactosamine methacrylamide)**	**PGalNAcMAm**
Poly(lactose methacrylamide)	PLacMAm-1
**Poly(lactose methacrylamide)**	**PLacMAm-2**
**Positively Charged Glycopolymers**	
**Lactose (Lac) functionalized PEI**	**Lac-PEI-1.8k**
**Lac functionalized PEI**	**Lac-PEI-10k**
Lac functionalized PEI	Lac-PEI-25k
Lac functionalized PEI	Lac-PEI-750k
Maltose (Mal) functionalized PEI	Mal-PEI-750k
**Negatively Charged Glycopolymers**	
**Sulfated Poly(lactose methacrylamide)**	**S-Lac-1**
**Sulfated Poly(lactose methacrylamide)**	**S-Lac-2**
Sulfated Poly(lactose methacrylamide)	S-Lac-3

**Table 3:** List of chemicals for synthetization of glycopolymers.

*N*-Acetyl-D-galactosamine (GalNAc; ≥99%)	Carbosynth, Compton, UK
d-lactose monohydrate (Lac; ≥96%)	Carbosynth
Methacryloyl chloride (purum, dist., ≥97%)	Sigma-Aldrich, St. Louis, MO, USA
Polyethyleneimine solution (branched, 50 wt.%, average Mn 60,000 by GPC, average Mw 750,000 by LS)	Sigma-Aldrich
Silica gel (high purity grade, pore size 60 Å, *N*,*N*,-dimethylformamide (≥99%)	Sigma-Aldrich
4,4′-azobis(4-cyanovaleric acid) (≥98%)	Sigma-Aldrich
Ammonium carbonate ((NH_4_)_2_CO_3_; ≥30.5% NH_3_, extra pure)	Carl Roth, Karlsruhe, Germany
Sodium carbonate (Na_2_CO_3_; ≥99%, anhydrous)	Carl Roth
Acetonitrile (ACN; ≥99.8%, for preparative HPLC)	Carl Roth
Methanol (MeOH; ≥98.8%)	VWR, Radnor, PA, USA
Deuterium oxide	VWR
Diethyl ether (Et_2_O; p. a.)	Chemsolute, Renningen, Germany
Tetrahydrofuran (THF)	Chemsolute
Chlorosulfonic acid (97%)	Acros Organics, Geel, Belgium

**Table 4:** Synthesis details of the reductive amination.

	Lac-PEI-1.8k	Lac-PEI-10k	Lac-PEI-25k	Lac-PEI-750k	Mal-PEI-750k
Lactose monohydrate per monomer unit	8 equiv	8 equiv	8 equiv	7 equiv	7 equiv
NaCNBH_3_ per Lac	5.0 equiv	5.0 equiv	5.0 equiv	3.5 equiv	3.5 equiv

**Table 5:** List of primary and secondary antibodies.

Target Protein	Animal	Clone	Manufacturer	Catalog Number	Dilution
Ki-67	Rabbit	Polyclonal	Abcam, Cambridge, UK	ab15580	1:100
Pax6	Rabbit	Polyclonal	Abcam	ab5790	1:50
BCRP/ABCG2	Mouse	BXP-21	Abcam	ab3380	1:50
Cy2	Donkey	Polyclonal	Jackson ImmunoResearch, West Grove, PA, USA	711-225-152	1:50
Cy3	Donkey	Polyclonal	Jackson ImmunoResearch	715-165-151	1:100

## Data Availability

The data presented in this study are openly available in [OSF] at [DOI 10.17605/OSF.IO/8F4W5]. The pictures from microscopy are available on request from the corresponding author.

## Supplementary Materials

The following supporting information can be downloaded at: https://www.mdpi.com/article/10.3390/ijms241814150/s1.
