# Supplementary material for: The Biocompatibility Analysis of Artificial Mucin-Like Glycopolymers
Source: Int J Mol Sci. 2023 Sep 15;24(18):14150. doi: 10.3390/ijms241814150 (PMC10532372; doi:10.3390/ijms241814150)
Supplement: Supplementary file 1 [file ijms-24-14150-s001.zip › ijms-2573575-supplementary.pdf]

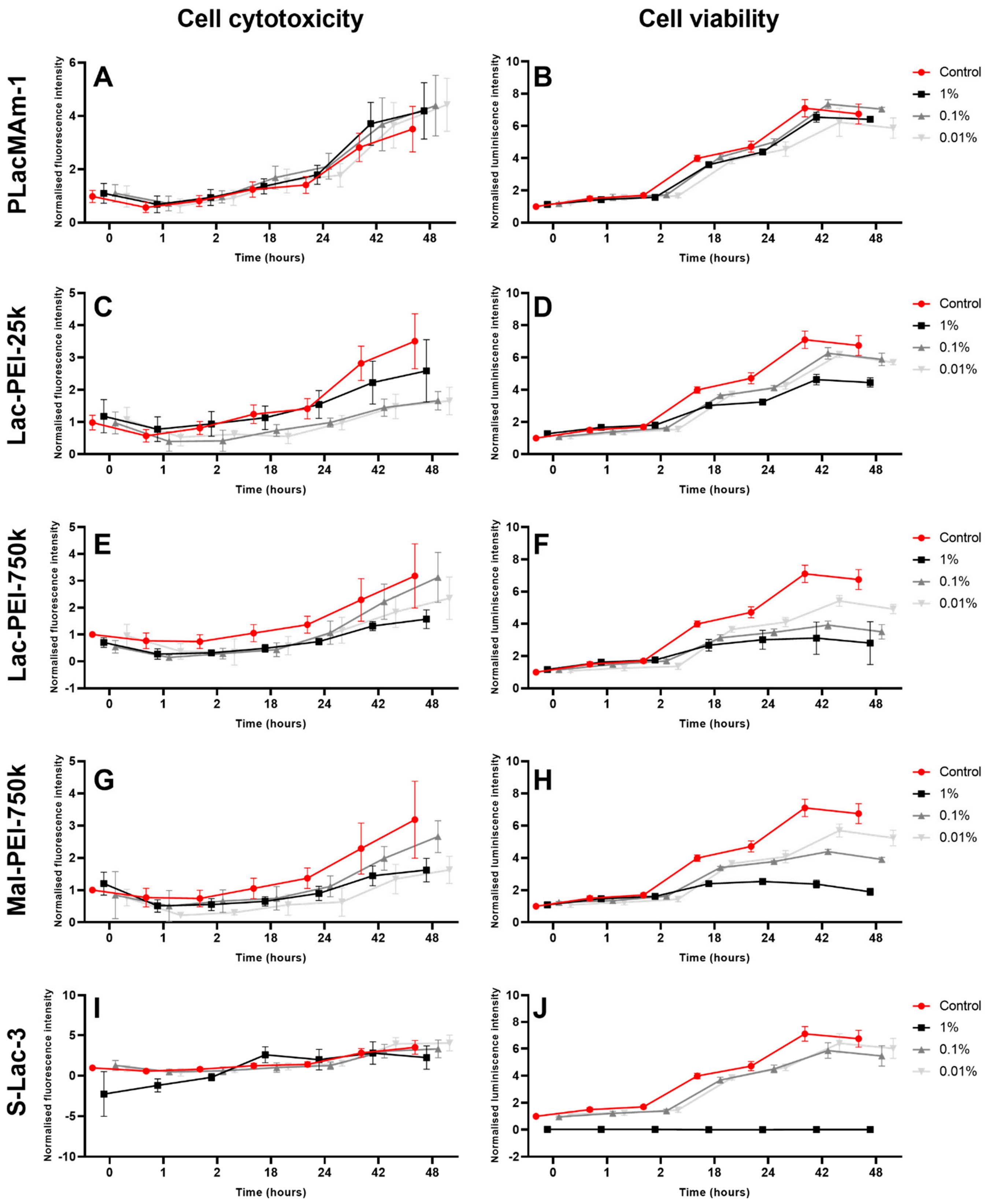

Figure S1. The cytotoxicity (A, C, E, G, I) and viability (B, D, F, H, J) assays of cultured HCE itself (Control), with different glycopolymers (in 1, 0.1, and 0.01% w/v concentration) and PEI (MW 750k). The intensity of luminescence (viability; y axis) and fluorescence (cytotoxicity; y-axis) were measured and at various time points (0-48h; x-axis) by Microplate Reader. The data were normalised to Control at time point 0. Interleaved symbols were used for every time point in the graphs.

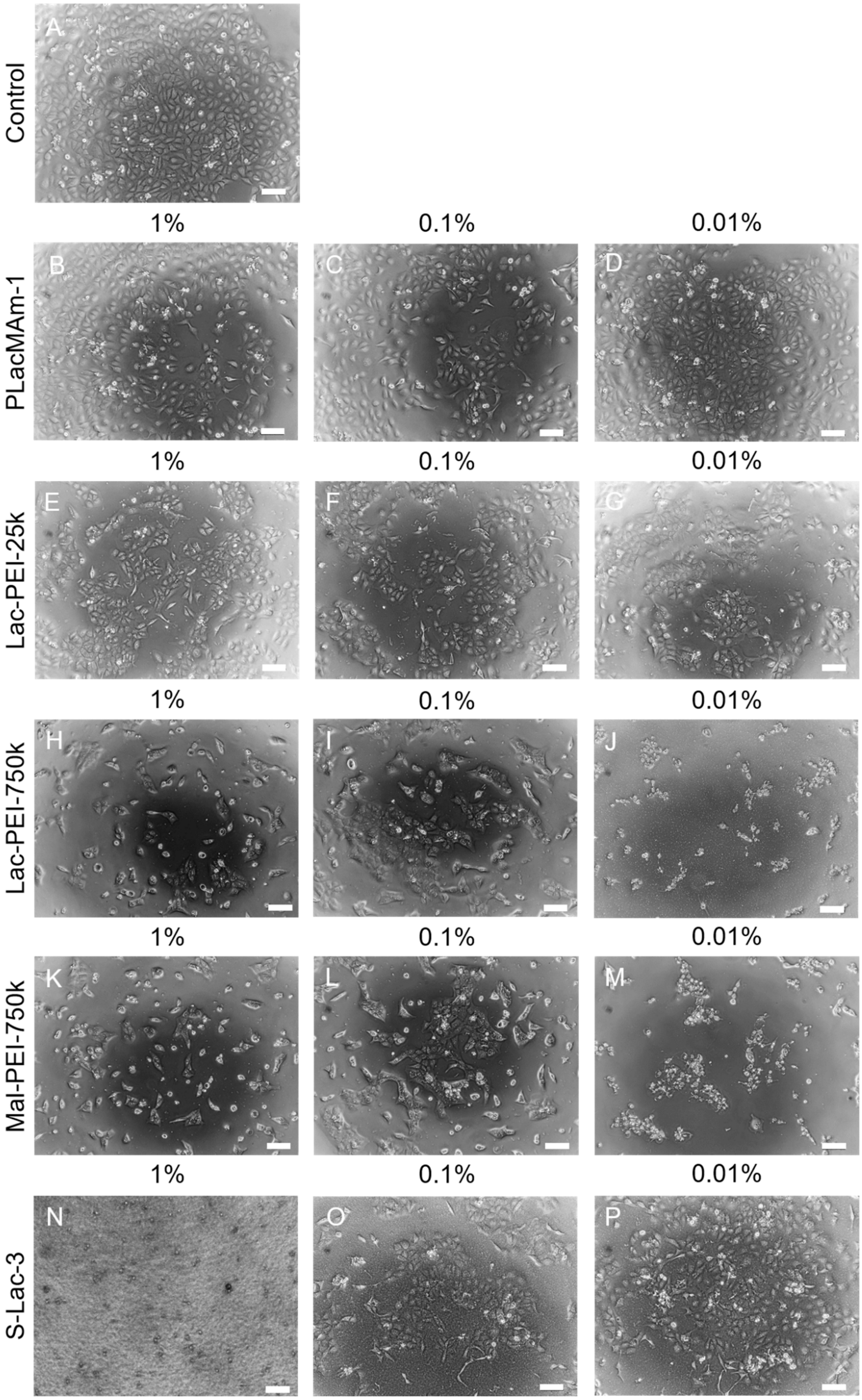

Figure S2. Morphology of cultured HCE itself (Control), with different glycopolymers (in 1, 0.1 and 0.01% w/v concentration) and PEI after 48h. Light microscopy, scale bars represent 100 μm.

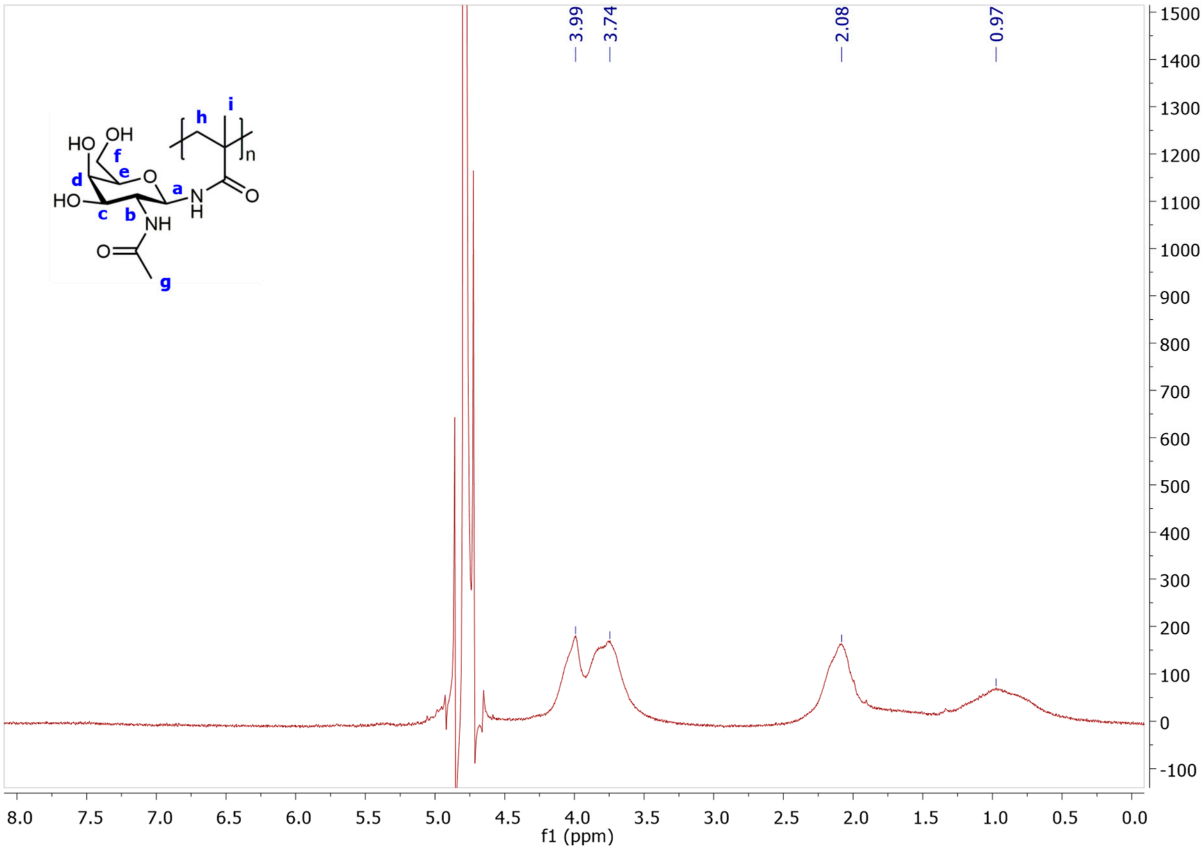

Figure S3. ^{1}H NMR spectrum of PGalNAcMAm.

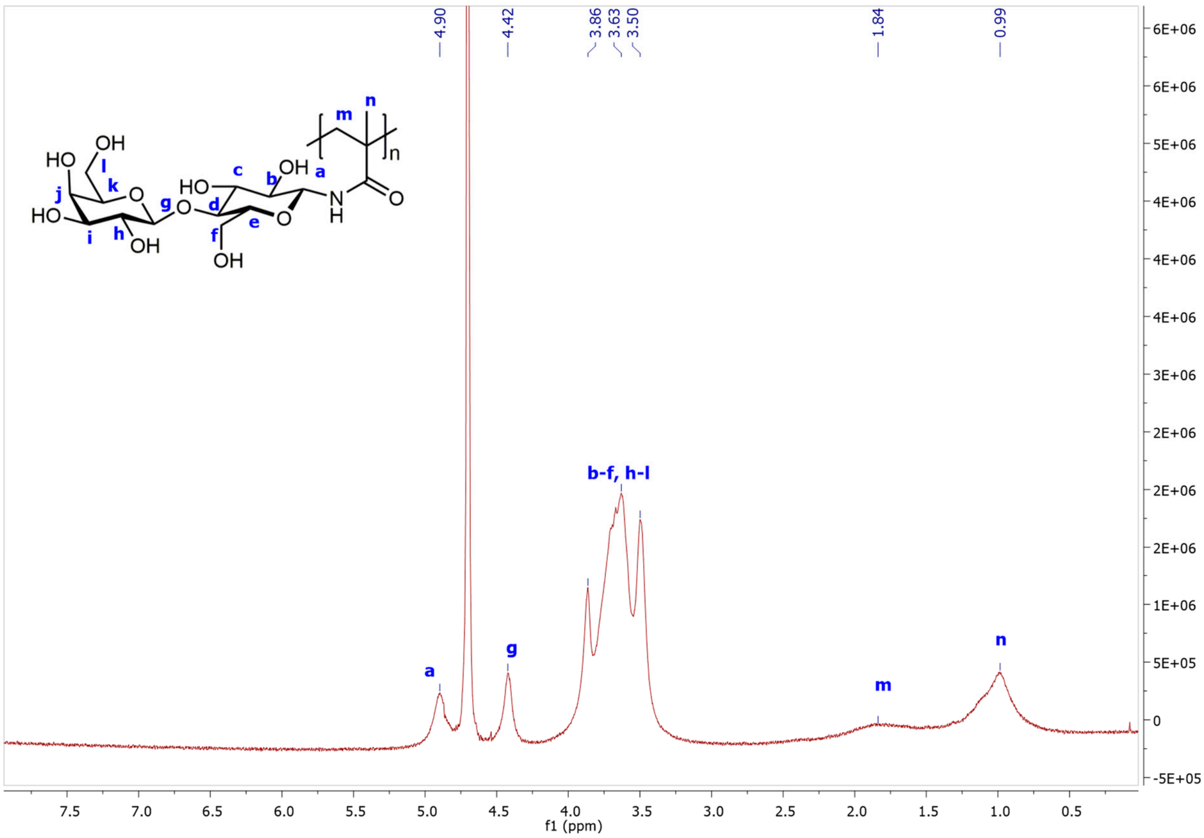

Figure S4. ^{1}H NMR spectrum of PLacMAm-1.

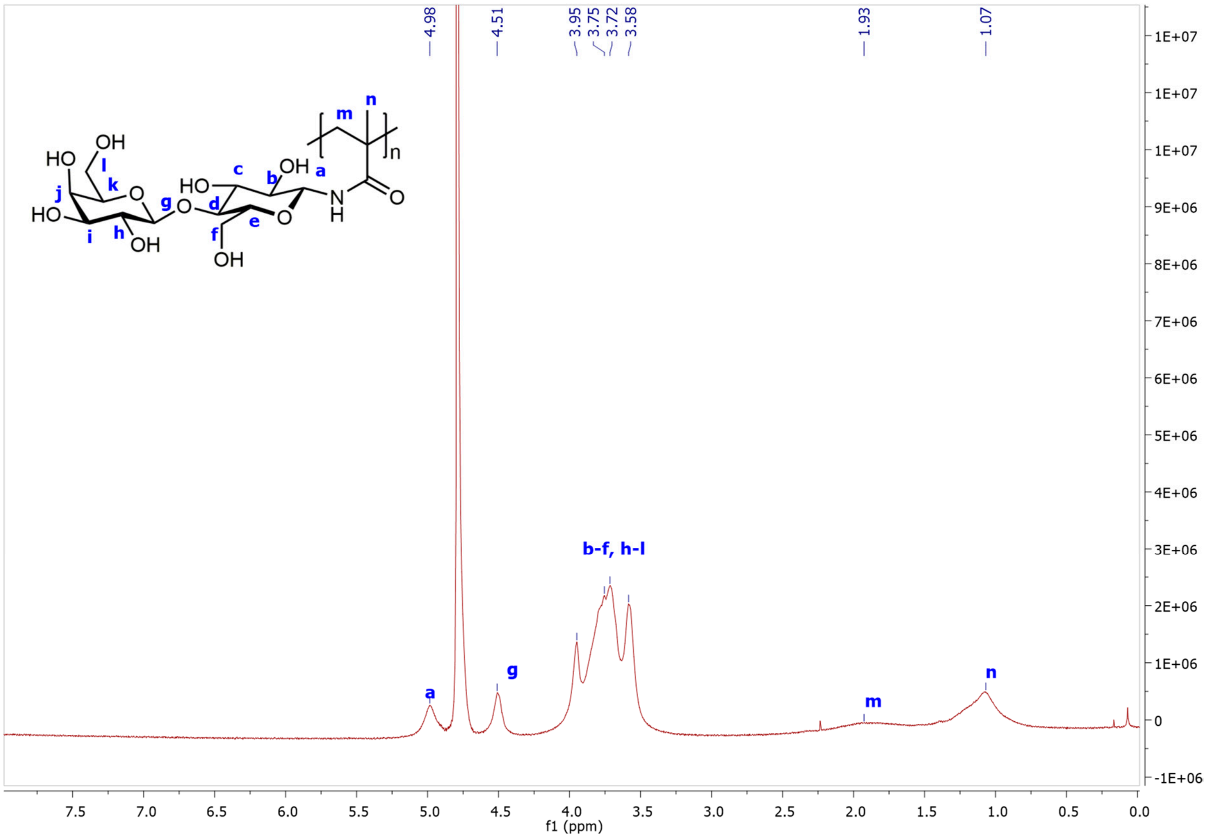

Figure S5. ^{1}H NMR spectrum of PLacMAm-2.

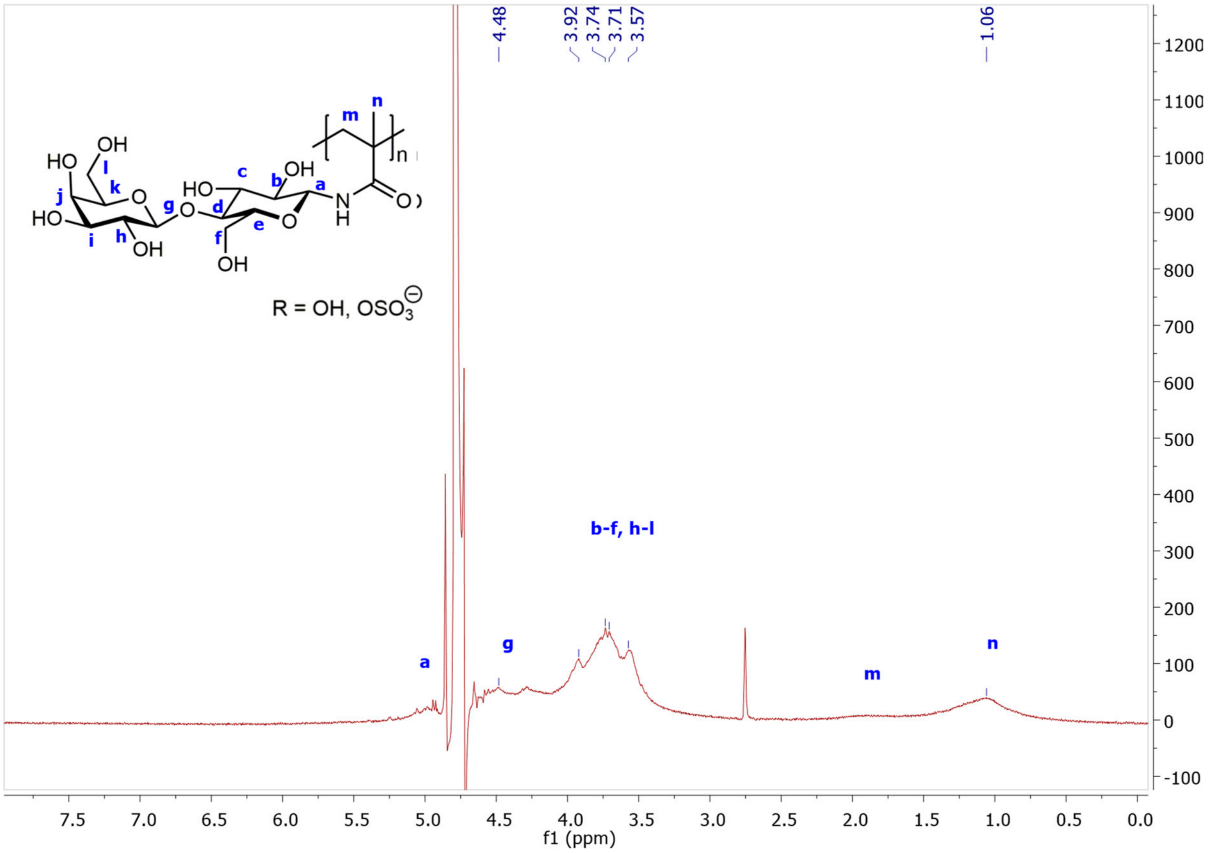

Figure S6. ^{1}H NMR spectrum of S-Lac-1.

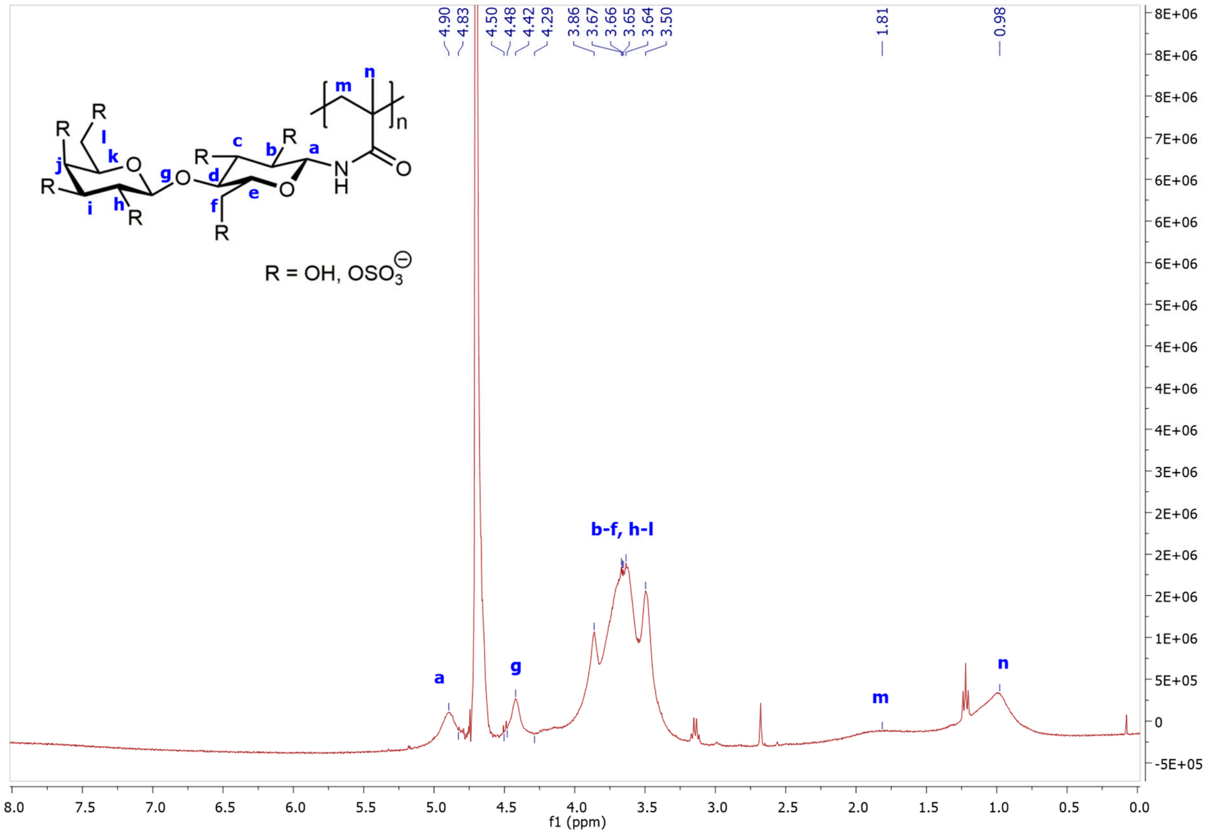

Figure S7. ^{1}H NMR spectrum of S-Lac-2.

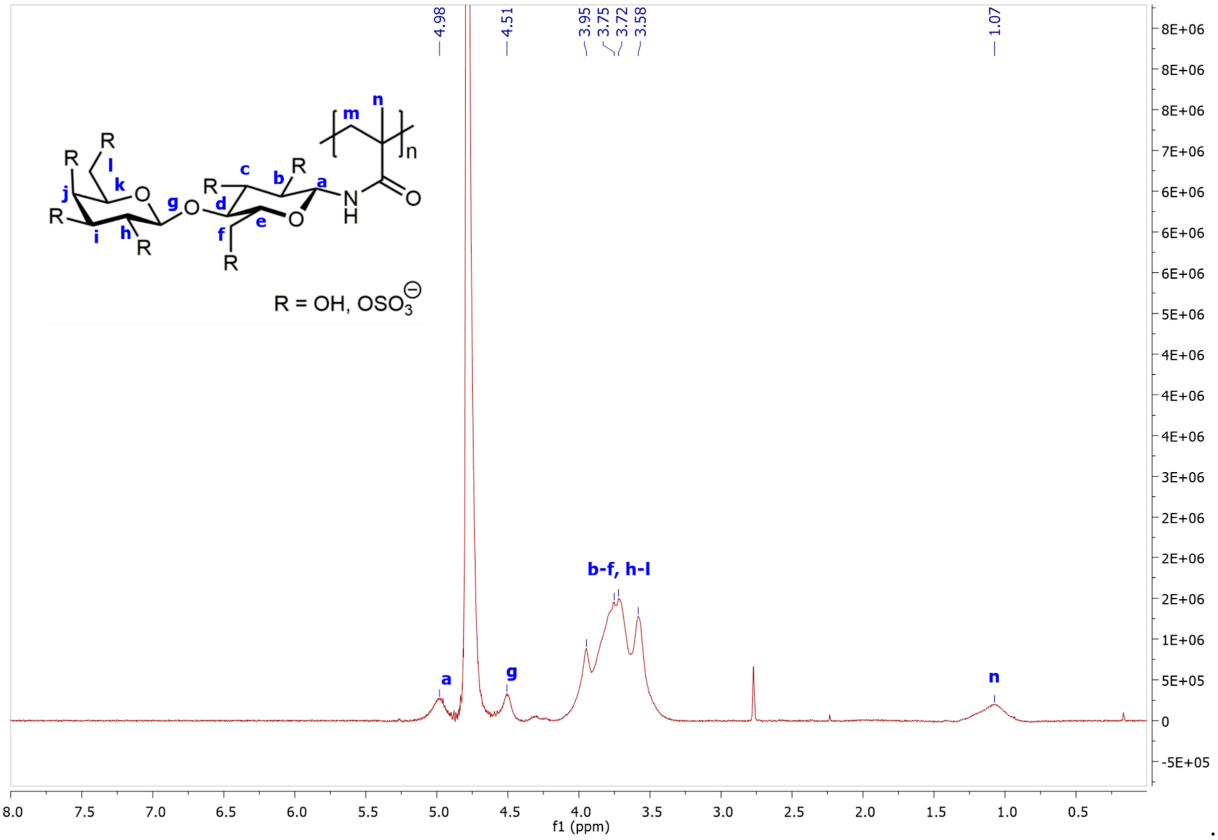

.

Figure S8. ^{1}H NMR spectrum of S-Lac-3. .

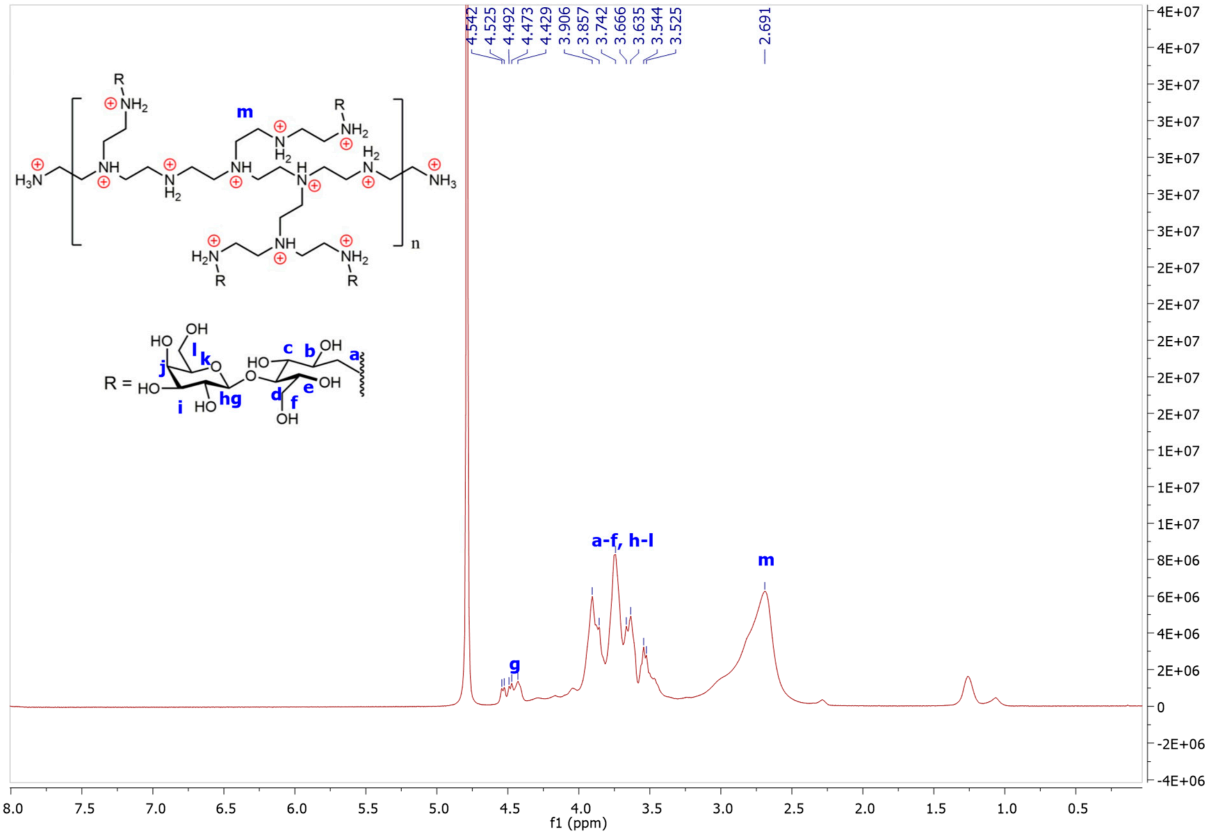

Figure S9. ^{1}H NMR spectrum of Lac-PEI-1.8k.

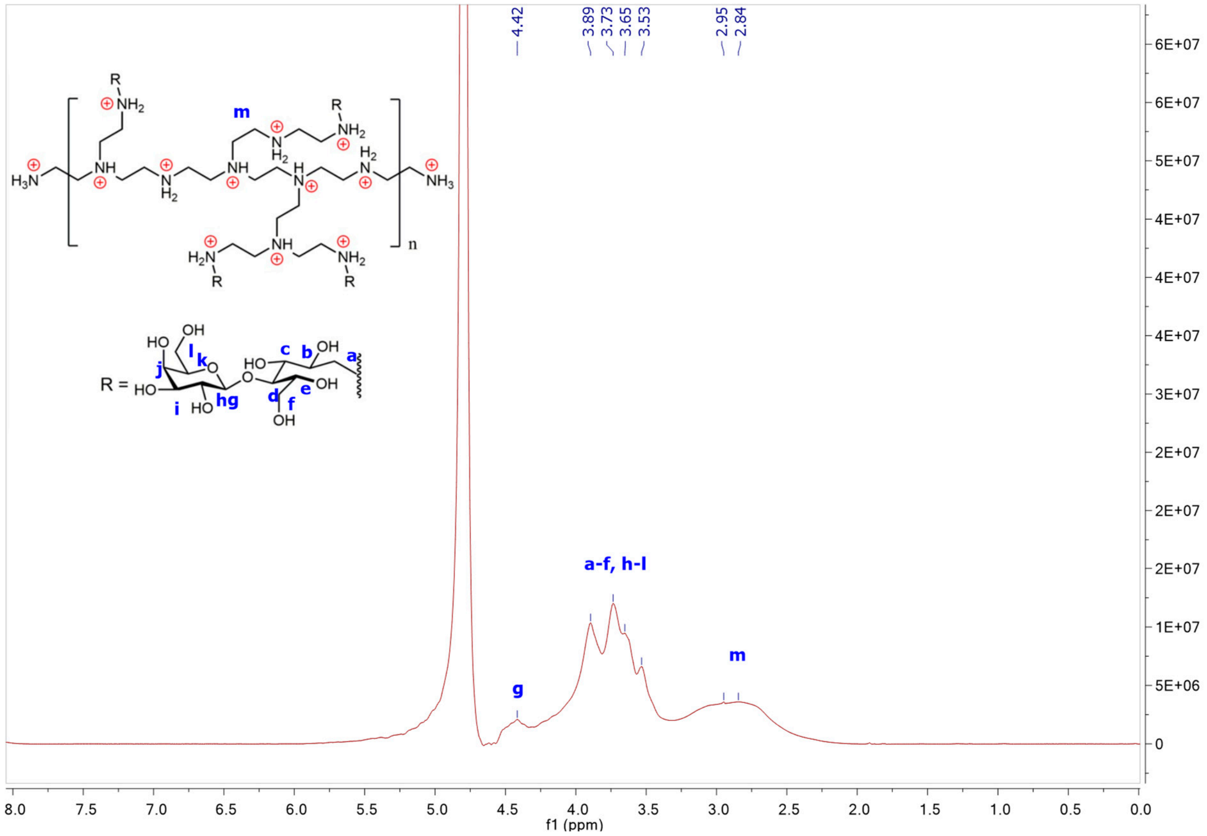

Figure S10. ^{1}H NMR spectrum of Lac-PEI-10k.

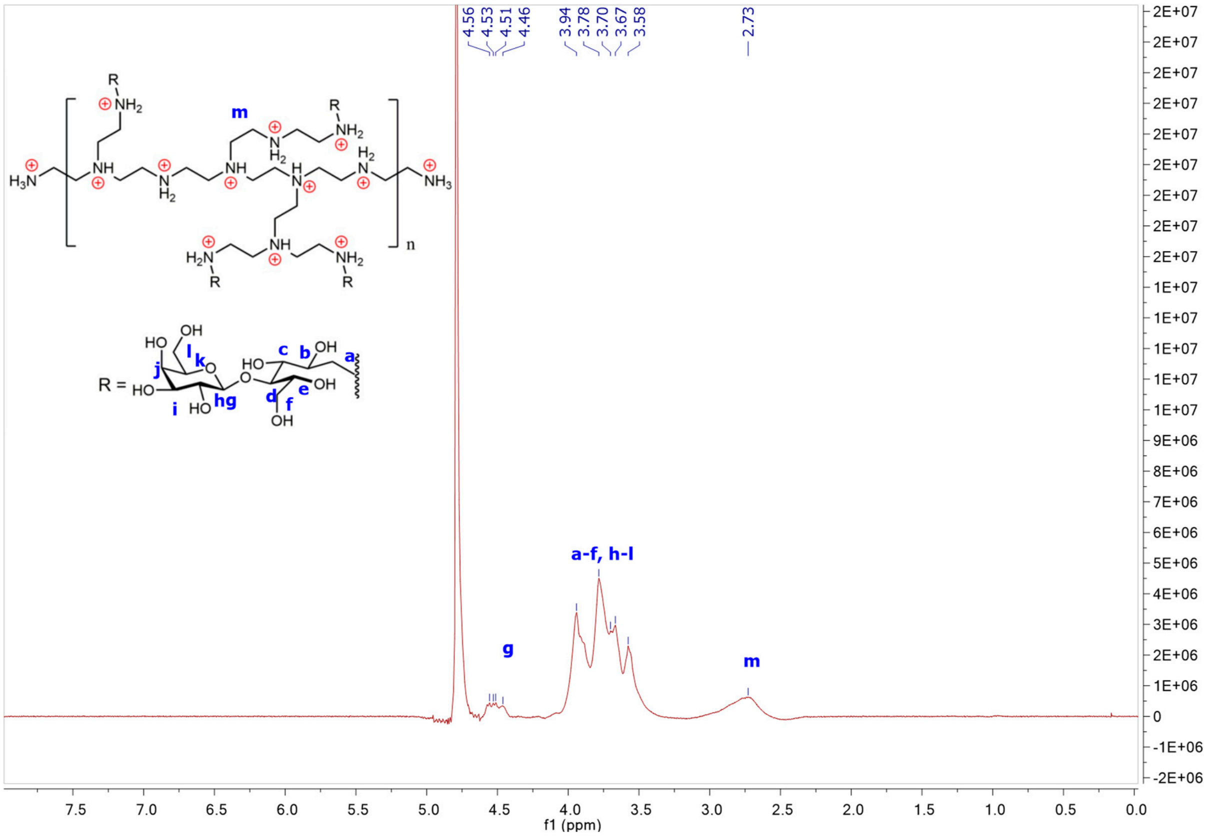

Figure S11. ^{1}H NMR spectrum of Lac-PEI-25k.

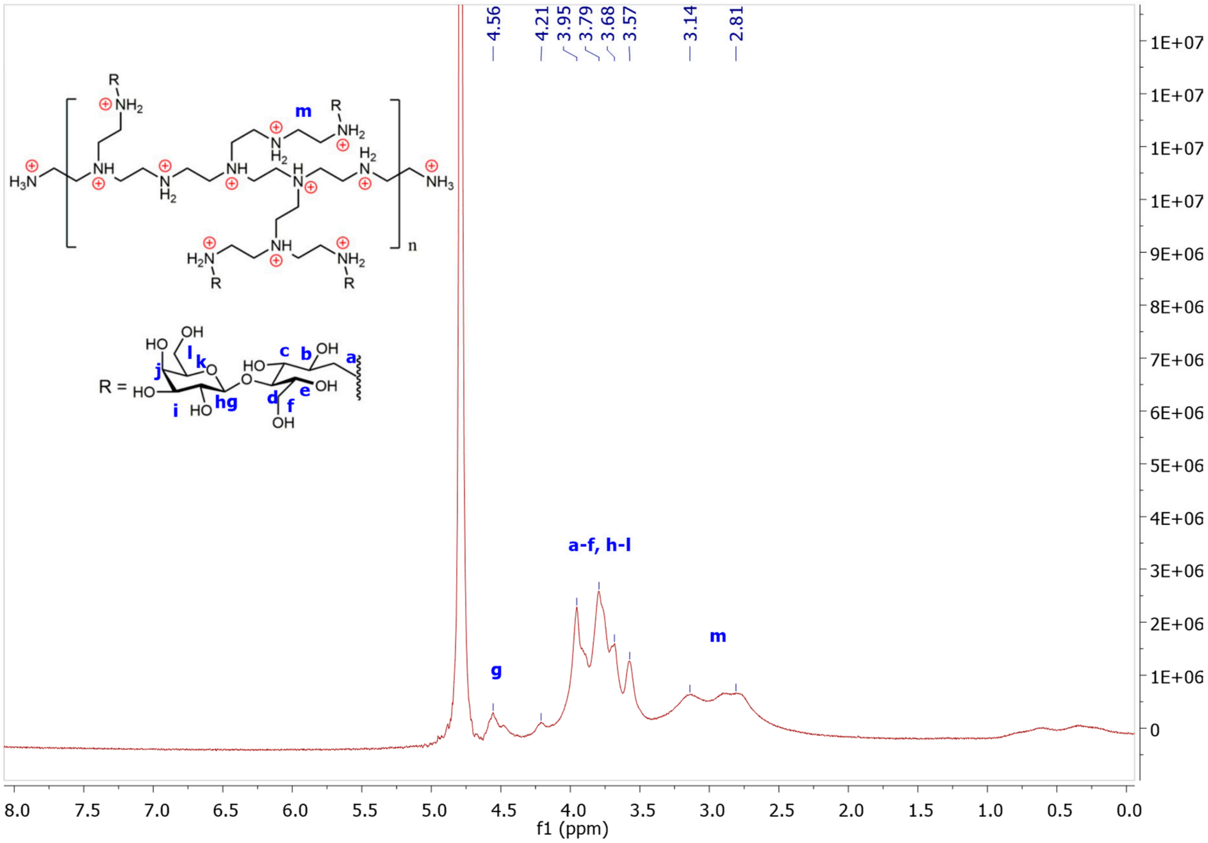

Figure S12. ^{1}H NMR spectrum of Lac-PEI-750k.

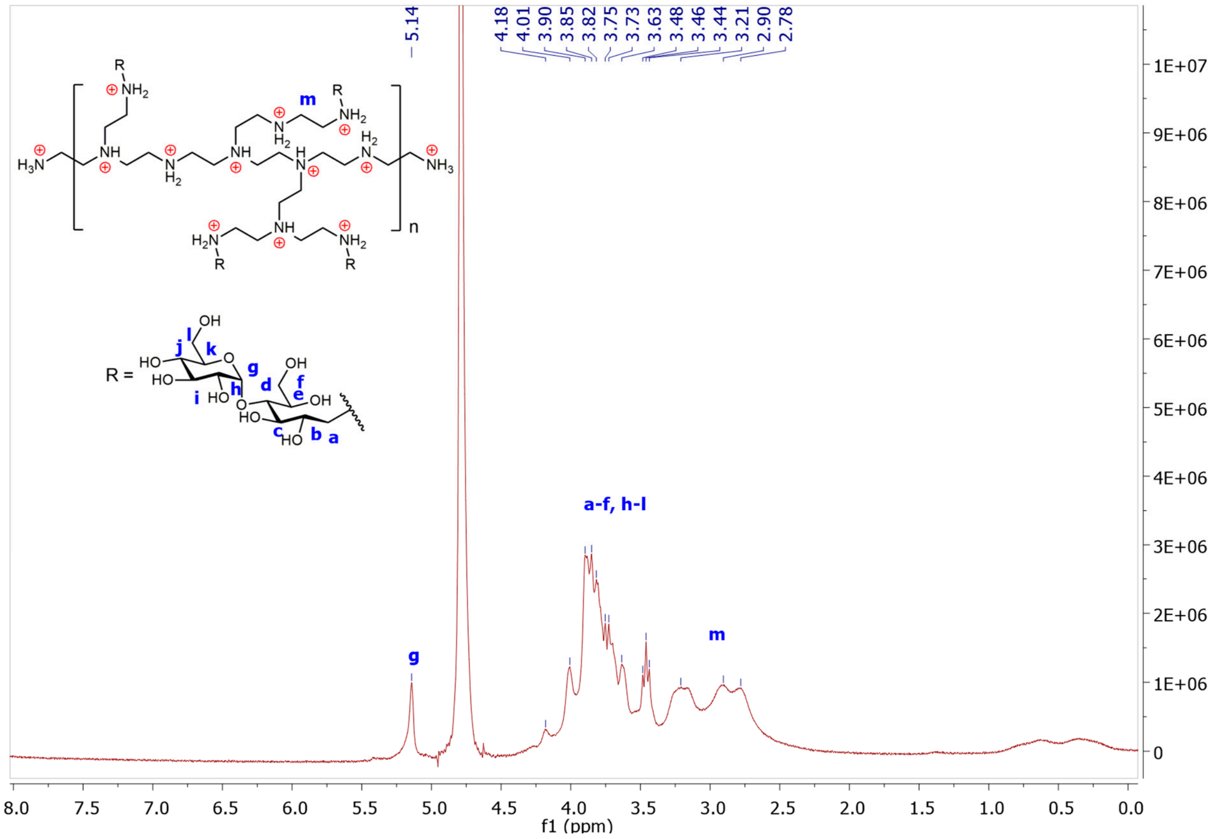

Figure S13. ^{1}H NMR spectrum of Mal-PEI-750k.
